# Redox Regulation of Metabolic Syndrome: From Biochemical Mechanisms to Nutritional Interventions

**DOI:** 10.3390/antiox10050638

**Published:** 2021-04-22

**Authors:** Mario Allegra

**Affiliations:** Department of Biological, Chemical and Pharmaceutical Sciences and Technologies, Università degli Studi di Palermo, Via Archirafi, 28, 90123 Palermo, Italy; mario.allegra@unipa.it; Tel.: +39-091-238-96803

According to its “harmonized” definition, metabolic syndrome (MetS) is described as a cluster of metabolic factors that increases the risk of cardiovascular diseases, diabetes (DM) and associated morbidities such as dementia. More specifically, the combined occurrence of at least three of the following five risk factors would qualify a person for MetS: hyperglycemia, hypertriglyceridemia, low high-density lipoprotein (HDL) cholesterol levels, hypertension and abdominal obesity. MetS can be modulated by genetics and epigenetics factors, fostered by sedentary lifestyle, influenced by the quality of food and sleep and controlled by gut microbiota. It has been estimated that more than one in three adults in the United States, and approximately 40% of people over the age of 40, have MetS, making it a major health hazard of the modern world [1].

From a mechanistic perspective, finely tuned, redox-dependent signal transduction mechanisms underlie and control metabolic dysregulations. Moreover, low-grade systemic inflammation is usually associated with such endocellular redox alterations and eventually leads to a dysfunctional metabolic rewiring [2].

Nutrition is one of the most important modifiable factors affecting human health. Appropriate dietary patterns have, indeed, been demonstrated to effectively counteract the immunometabolic alterations leading to MetS development [3]. Along these lines, this Special Issue has been devoted to gathering novel, relevant and significant contributions on the redox-dependent biochemical mechanisms underlying MetS, along with nutritional studies analyzing the impact of diet on this metabolic disorder. New information has been added to this field by means of 14 articles, with 10 original papers and 4 reviews.

In the first paper, Valenzano et al. have evaluated the effect of a very low-calorie ketogenic diet (VLCKD) in a population of obese patients. The study drew inspiration from the current knowledge on the effects of caloric restriction and dietary ketosis on the reduction of visceral adipose tissue (VAT). By employing an integrated in vivo and ex vivo experimental approach, the authors demonstrated that VLCKD was able to decrease VAT, ameliorating adiposity and blood chemistry parameters. Moreover, the short-term mild dietary ketosis did not appear to have a cytotoxic effect, nor was it a condition able to increase oxidative stress. Relevantly, the study also demonstrated for the first time an effect of the VLCKD upon the orexinergic system. As a whole, these data further support the usefulness of such a therapeutic dietary intervention to promote obesity reduction via hypothalamic mechanisms [4].

In the light of the strict interconnections between the Mediterranean diet, epigenetics and inflammation, Bordoni et al. investigated the nutrigenomic properties of two high-quality oils, extra virgin olive (EVO) oil and that of *Nigella sativa* (NG), in an in vitro model of low-grade inflammation in human macrophages. The authors demonstrated that both EVO and NG oil reduced low-grade inflammation through antioxidant and epigenetic mechanisms. Indeed, both oils were able to restore normal expression levels of DNMT3A and HDAC1 (but not DNMT3B), which were altered under inflammatory conditions. Moreover, EVO oil was able to prevent the increase in TET2 expression and reduce global DNA methylation that were measured in inflamed cells. Interestingly, NG oil seemed to be more efficient in the control of proinflammatory cytokines, whereas EVO oil better helped to counteract redox imbalance [5].

It is now well established that dietary interventions can exert positive effects on MetS and also induce alterations of gut microbiota. Along these lines, Guirro et al. have assessed the effects of the citrus polyphenol hesperidin in an in vivo model of MetS with the aim to elucidate the host–microbiome interplay with extensive anthropometric and biochemical characterizations. Interestingly, the authors demonstrated that hesperidin supplementation ameliorated the plasma lipid profile, as well as blood pressure, insulin sensitivity and relevant markers of arterial stiffness and inflammation. Coherently, metabolomics also revealed an improvement of the lipidomic profile, a decrease in circulating amino acids and a lower excretion of inflammation- and oxidative stress-related metabolites. Relevantly, animals excreted larger amounts of microbial-derived metabolites, which positively correlated with the Bacteroidaceae family. As a whole, these data do support the hypothesis for a potential use of hesperidin as a prebiotic in the combo therapy of MetS [6].

Besides the beneficial effects of dietary patterns to prevent and/or counteract MetS development, supplementation with plant-origin extracts has been widely explored over the last decade. Within this remit of intense research, the study from de la Fuente-Fernandez et al. has analyzed the protective effects of a carob fruit extract (CSAT+®) on the cardiometabolic alterations associated with MetS in mice. The authors discovered that CSAT+® supplementation was able to ameliorate both the glucidic and lipidic plasma profile, significantly reducing peripheral insulin resistance (IR), inflammation and oxidative stress. Moreover, CSAT+® treatment also prevented MetS-induced hypertension, improved vascular response and attenuated endothelial dysfunction (ED). Finally, cardiomyocyte apoptosis and an ischemia–reperfusion-induced decrease in cardiac contractility was also prevented by the fruit extract supplementation [7]. 

A key issue in the development of MetS is the difference between metabolically healthy obesity (MHO) and metabolically unhealthy obesity (MUO) phenotypes, partly attributable to genetic traits that modulate body fat distribution and obesity-related metabolic aspects. Within this context, no solid reports are available on the genotypic effect of the leptin receptor (LEPR) gene on fat distribution and obesity-related metabolic abnormalities in the Korean population. Along these lines, the study by Jang et al. evaluated whether the difference between MHO and MUO phenotypes might be partly attributable to the LEPR rs8179183 polymorphism in obese Korean females. Interestingly, the results of the work revealed that LEPR rs8179183 was associated with metabolic health status in a Korean population with obesity. Moreover, the paper reported on MUO individuals with the GG genotype having the most unfavorable values of glucose-related markers, lipid profiles, adipokines, oxidative stress markers and regional fat distribution [8].

Epidemiological data clearly show that, within dietary patterns and lifestyles, sugar-sweetened beverages (SSBs) are deeply implicated in MetS development. In light of scanty mechanistic insights underlying this process, Benade et al. have established a unique rat model of long-term SSB consumption to deeply analyze the link between SSB consumption and the onset of type 2 diabetes mellitus (T2DM). Interestingly, the authors showed that SSB consumption increased hepatic ER stress and altered hepatic mitochondrial dynamics, antioxidant response and calcium handling across mitochondria-associated ER membranes. Relevantly, the authors underlined that at this relatively early stage of SSB intake, the liver could be able to initiate adaptive responses to significantly counteract such stressors. However, the observed increased mitochondrial fission and decreased fusion could represent a possible mechanism responsible for the development of MetS and T2DM in the longer term [9].

Nitric oxide (NO) is widely reported to play a pivotal role in cardiovascular and metabolic regulations. Coherently, nitrite, a precursor of the NO reservoir abundant in green leafy vegetables, has been proposed to exert protective effects against T2DM and cardiovascular diseases. On the other hand, while the role of iNOS as a pro-inflammatory agent is well established, its protective role under normal physiological conditions and cardiovascular disorders is less well investigated. Along these lines, the work by Aggarwal et al. investigated the incidence of IR in chow-fed iNOS^−/−^ mice and the effect of nitrite supplementation on the rescue of systemic, hepatic and adipose tissue insulin sensitivity. Interestingly enough, the authors demonstrated that chow-fed adult iNOS^−/−^ mice exhibited systemic IR, dyslipidemia and metabolic perturbations. Relevantly, nitrite supplementation significantly counteracted IR and glucose tolerance and reduced lipid accumulation by improving hepatic insulin sensitivity and glucose and lipid homeostasis. While demonstrating that nitrite supplementation improves insulin sensitivity and metabolic homeostasis, the authors further highlight the metabolic role of iNOS [10].

It has been demonstrated that NO, together with glutathione (GSH), is involved in the modulation of the hepatic regulation of glucose metabolism. Taking into account the incomplete current knowledge of the metabolic action of GSH and NO in glucose homeostasis, the study from Sousa-Lima et al. was designed to evaluate whether treatment with S-nitrosoglutathione (GSNO) could revert high-sucrose diet-induced IR in vivo. Interestingly, GSNO administration was able to revert IR induced by sucrose feeding in a dose-dependent manner, demonstrating an insulin-sensitizing effect in vivo. From a mechanistic perspective, these effects were associated with an increased expression of both insulin receptors and p-Akt in muscle cells after GSNO treatment. In line with this, the authors suggested that GSNO may act as a potential pharmacological tool for the treatment of IR in obesity and T2DM [11].

T2DM-induced hyperglycemia has been demonstrated to lead to oxidative stress-mediated fibrosis in different tissues and organs. The endothelial-to-mesenchymal transition (EndMT) has been proposed to play a role in T2DM-associated fibrotic conditions. Along these lines, Giordo et al. have investigated whether EndMT is implicated in the diabetic retinopathy fibrotic process and evaluated if resveratrol could counteract EndMT in vitro. Interestingly, the authors discovered that the treatment of primary human retinal endothelial cells (HRECs) with high glucose (HG) resulted in an increase in intracellular ROS levels and fostered phenotype shifting towards EndMT. The mechanism appeared to be mediated by a PKC involvement leading to NADPH-associated ROS generation. Interestingly, resveratrol was able to counteract EndMT via a downregulation of NADPH oxidase, suggesting a resveratrol-based potential protective therapy to prevent diabetic-associated complications [12].

Childhood obesity and its metabolic complications have been related to deficient antioxidant capacity and oxidative stress. Erythrocytes (RBCs) are constantly exposed to oxidative stress and hence are provided with powerful antioxidant systems strongly depending on the pentose phosphate pathway (PPP). Along these lines, in their work, Gonzalez-Dominguez et al. aimed to assess the efficacy of the PPP (through its NADP dehydrogenases) to provide reducing power in the RBCs of obese children with IR as compared to a group of metabolically healthy obese participants and a group of healthy controls. Interestingly, the authors demonstrated that obese children with IR present higher levels of oxidative damage, blunted capacity to generate reducing power and hampered function of key NADPH-dependent antioxidant enzymes. This condition resulted in a deficient response to a glucose overload as compared with the other study groups. Interestingly enough, the authors found a physiological deviation of redox balance (oxidative eustress) in obese children without IR and a supraphysiological deviation (oxidative distress) in the IR group [13].

It has widely been recognized how IR and T2DM can play a key role in the etiopathogenesis of neurodegenerative pathologies. Additonally, an increasing number of nutraceuticals have been evaluated for their potential to exert protective effects in this regard. Within this remit of research, Amato et al. reviewed experimental data on the ability of curcuminoids, silymarin, chlorogenic acid and Aphanizomenon flos-aquae-derived compounds to exert beneficial effects on neurodegeneration and in particular in Alzheimer’s disease models [14].

Hypomagnesemia is commonly observed in both cardiovascular diseases and several risk factors associated with MetS, such as T2DM and hypertension. Interestingly, low serum magnesium (Mg) has been proposed as a predictor for cardiovascular and all-cause mortality. In their review, Liu et al. reported on the possible mechanisms by which Mg deficiency could play detrimental roles in cardiovascular diseases and reviewed the results of clinical trials of Mg supplementation for heart failure, arrhythmias and other cardiovascular diseases [15]. 

Eryptosis is a coordinated, programmed cell death culminating in the disposal of cells without disruption of the cell membrane and the release of endocellular oxidative and pro-inflammatory milieu. While providing a convenient form of death for RBCs, dysregulated eryptosis may result in a series of detrimental and harmful pathological consequences. In light of the crucial role played by the eryptotic process in MetS-related endothelial dysfunction (ED), Restivo et al. reviewed the molecular mechanisms leading to eryptosis in MetS-related conditions (hyperglycemia, dyslipidemia, hypertension and obesity). Moreover, the authors also reported the clinical evidence supporting or proposing a role for eryptosis in ED associated with MetS cardiovascular complications [16].

MetS and non-alcoholic fatty liver disease (NAFLD) are two different entities sharing common clinical and physio-pathological features, with IR being the most relevant. In the last review, Rinaldi et al. reported on the “bad company” constituted by MS and NAFLD and discussed both the mechanistic aspects and the clinical impact related to these pathological conditions [17].
